# Noiseless photonic non-reciprocity via optically-induced magnetization

**DOI:** 10.1038/s41467-021-22597-z

**Published:** 2021-04-22

**Authors:** Xin-Xin Hu, Zhu-Bo Wang, Pengfei Zhang, Guang-Jie Chen, Yan-Lei Zhang, Gang Li, Xu-Bo Zou, Tiancai Zhang, Hong X. Tang, Chun-Hua Dong, Guang-Can Guo, Chang-Ling Zou

**Affiliations:** 1 CAS Key Laboratory of Quantum Information & CAS Center For Excellence in Quantum Information and Quantum Physics, University of Science and Technology of China, Hefei, P. R. China; 2 State Key Laboratory of Quantum Optics and Quantum Optics Devices, and Institute of Opto-Electronics, Shanxi University, Taiyuan, P. R. China; 3 Collaborative Innovation Center of Extreme Optics, Shanxi University, Taiyuan, P. R. China; 4 Deparment of Electric Engineering, Yale University, New Haven, CT USA

**Keywords:** Quantum optics, Single photons and quantum effects

## Abstract

The realization of optical non-reciprocity is crucial for many applications, and also of fundamental importance for manipulating and protecting the photons with desired time-reversal symmetry. Recently, various new mechanisms of magnetic-free non-reciprocity have been proposed and implemented, avoiding the limitation of the strong magnetic field imposed by the Faraday effect. However, due to the difficulties in separating the signal photons from the drive laser and the noise photons induced by the drive laser, these devices exhibit limited isolation performances and their quantum noise properties are rarely studied. Here, we demonstrate an approach of magnetic-free non-reciprocity by optically-induced magnetization in an atom ensemble. Excellent isolation (highest isolation ratio is $51.{5}_{-2.5}^{+6.5}\ {\rm{dB}}$) is observed over a power dynamic range of 7 orders of magnitude, with the noiseless property verified by quantum statistics measurements. The approach is applicable to other atoms and atom-like emitters, paving the way for future studies of integrated photonic non-reciprocal devices.

## Abstract (web summary)

Optical nonreciprocity through magneto-optical effects requires bulky apparatuses and strong magnetic fields, while magnetic-free approaches are difficult to implement. Here, the authors use optically-induced magnetisation in an atomic ensemble to get 50 dB of isolation over a large power dynamic range.

## Introduction

Lorentz reciprocity and its violation, associating with the time-reversal symmetry breaking, are of fundamental and conceptual importance in optics^1–3^, and have also led to controversies in the photonics research community^4^. In practical optical applications, non-reciprocal optical devices, including isolator, circulator, and gyrator, are indispensable and ubiquitous. The prominent mechanisms realizing the optical non-reciprocity are the magnetic circular dichroism and circular birefringence (Faraday effect) in bulky magneto-optical materials^5,6^. A strong magnetic bias field changes the dipole momentum or transition frequencies of dielectrics by inducing the Zeeman splitting of electron spin states and modifying the electronic wavefunctions, thus breaks the reciprocity of light^3^. However, the utilization of conventional Faraday-effect-based non-reciprocal devices is restricted in many scenarios. On one hand, the difficulties in processing the magneto-optical materials impose obstacles for fabricating high-performance optical micro-structures^7,8^. On the other hand, the strong bias magnetic field is incompatible with many other optical components, such as the spin ensembles and superconducting-photonic interface, thus prevents the applications of non-reciprocal devices in hybrid photonic integrated circuits^9,10^.

Over the past decade, great efforts have been dedicated to realizing magnetic-free non-reciprocity of light^3^. Ingenious ideas and new experimental techniques are developed to break the limit of the conventional Faraday effect, such as optical drive induced directional frequency conversion^11–14^, storage^15–17^ or amplification^18–21^, the synthetic magnetic field in a loop of coupled-resonator^22,23^, and RF/acoustic drive induced spatio-temporal modulation of refraction index^24–30^. The common principle behind these approaches is the orbital momentum conservation in the coherent mode conversions, which induces absorption or phase shift for photons input from a selected direction. However, such a mechanism imposes limitations for non-reciprocal devices. The momentum conservation in the coherent mode conversions usually requires stringent phase-matching conditions, and the strong drive will induce inevitable background noise photons due to the nonlinear parametric processes. In addition, the low-energy excitations, such as the mechanical mode involved in the optomechanical interactions, require the drive laser frequency to be at most few GHz detuned from the signal frequency and also generate noise photons due to the thermal excitations, imposing great difficulties in separating the weak signals from backgrounds. As a result, although great progress has been achieved in the magnetic-free optical isolation^12–14,16–18,27–32^, the conservation of quantum properties of signals transmitted through the isolator has not been verified experimentally.

Here, we propose and demonstrate a scheme to realize the non-reciprocity via the optically-induced magnetization (OIM) of an atomic medium. Isolation ratios of $30.{3}_{-0.2}^{+0.3}\ {\rm{dB}}$ and $51.{5}_{-2.5}^{+6.5}\ {\rm{dB}}$ are realized with a warm atom vapor in free space and in a traveling-wave cavity, respectively. Dispersive non-reciprocal effects analogous to Faraday effect are also observed with a non-reciprocal optical mode frequency splitting exceeding 100 MHz. It is worth noting that the non-reciprocal photon-atom interaction was investigated previously^33,34^, however, under the requirement of tens of Gauss bias magnetic field. The magnetic-free OIM mechanism is distinct by treating the atom ensemble as a magnetizable dielectric medium, where incoherent population transfer could be utilized for achieving the non-reciprocity. Its crucial advantages include that the optical drive frequency could be far detuned from the signal, avoiding the difficulty associated with phase matching and drive filtration, robustness against drive fluctuations, and elimination of drive-induced noises at signal frequencies. We demonstrate the preservation of the signal’s quantum statistic feature, proving the inherent noiseless property of this mechanism. We believe that our experiments could stimulate further experimental and theoretical efforts on OIM, to demonstrate non-reciprocal devices in photonic integrated circuits, and find applications in quantum photonic chips and explore intriguing topological properties of light^35^.

## Results

### Principle of OIM-based non-reciprocity

**Fig. 1:**
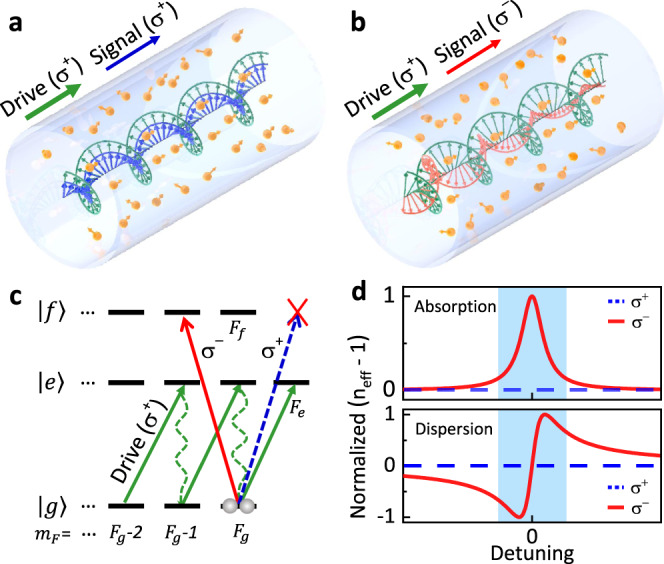
Schematic of circular birefringence and circular dichroism arising from optically-induced magnetization. **a**, **b** In an atomic medium with non-zero magnetization induced by a *σ*^+^-polarized drive field, the *σ*^+^- and *σ*^−^-polarized signals propagate at different velocities and experience different absorption losses. **c** The energy diagram of typical atoms or atomic-like emitters, with degenerate ground Zeeman levels ($\left|g\right\rangle$) and the allowing optical transitions to excited energy levels ($\left|e\right\rangle$, $\left|f\right\rangle$). The drive field ($\left|g\right\rangle \to \left|e\right\rangle$) induces the magnetization of the atoms as the population concentrates to one side of the Zeeman levels, which leads to the different response to *σ*^+^- and *σ*^−^-polarized signals ($\left|g\right\rangle \to \left|f\right\rangle$) due to selection rules. **d** The illustration of absorption and dispersion in a magnetized atomic medium for *σ*^+^- and *σ*^−^-polarized signals. Absorption dominates in the shadow region where the signal is near-resonant to the atomic transitions, while dispersion dominates for off-resonant cases.

Figure 1(a) and (b) schematically illustrates the general principle of the noiseless all-optical non-reciprocity that is based on the OIM. In the presence of an external circularly-polarized drive field, the optical response of an atom ensemble to a weak signal field is modified, leading to a circular-polarization dependent propagation velocity and absorption. As indicated in Fig. 1(c), the atoms possess hyperfine ground spin states $\left|g,{m}_{F}\right\rangle$, with *m*_*F*_ denoting the quantum spin number with $\left|{m}_{F}\right|\le {F}_{g}$. By introducing the ancillary energy level $\left|e\right\rangle$ with *F*_*e*_ ≥ *F*_*g*_, the drive laser with the *σ*^+^ polarization changes the population of the atoms to *m*_*F*_ = *F*_*g*_, and thus builds up an effective magnetization of the atom spin states (green lines in Fig. 1(c)). For an input signal near-resonant to $\left|g\right\rangle \to \left|f\right\rangle$ by *F*_*f*_ ≤ *F*_*g*_, the drive laser could transfer the population to *m*_*F*_ = *F*_*g*_, for which the its *σ*^+^-transition is forbidden (blue dash line in Fig. 1(c)). Therefore, the atomic medium in Fig. 1(a) is transparent to the *σ*^+^-polarized signal. In contrast, the transition for the *σ*^−^-polarized signal is allowed (red solid line in Fig. 1(c)), thus the signal attenuates when propagating  in the atomic media with the configuration shown in Fig. 1(b).

Figure 1(d) illustrates the OIM-induced circular birefringence and dichroism, as the polarized medium induces velocity and absorption change to the probe signals. When the circularly polarized probe signal is off-resonant to the atoms (with detuning Δ), the atoms induce effective phase change ∝ 1/Δ while the absorption is suppressed to ∝ 1/Δ^2^. Different from the conventional Faraday effect, where the external magnetic field induces the Zeeman energy level shifts and thereby circular birefringence, our scheme bases on ground state magnetization of the atoms induced by circularly polarized drive. The scheme is also distinct from previous nonlinear optical schemes^11–20^, where the phase-matching condition breaks the time-reversal symmetry of light. The drive field could be applied to any ancillary transition that couples to the target ground levels and is not necessary to be coherent with the signal. Therefore, the scheme allows broad bandwidth non-reciprocity beyond the limitation of phase-matching, permits more convenient filtration of drive laser, and the device would not induce noises for signals. It is also noted that the population condition illustrated in Fig. 1(c) is not strictly required for realizing non-reciprocity, because the time-reversal symmetry could be achieved as long as the uniform population distribution over all *m*_*F*_ states is broken to produce nonzero net spin polarization.

**Fig. 2:**
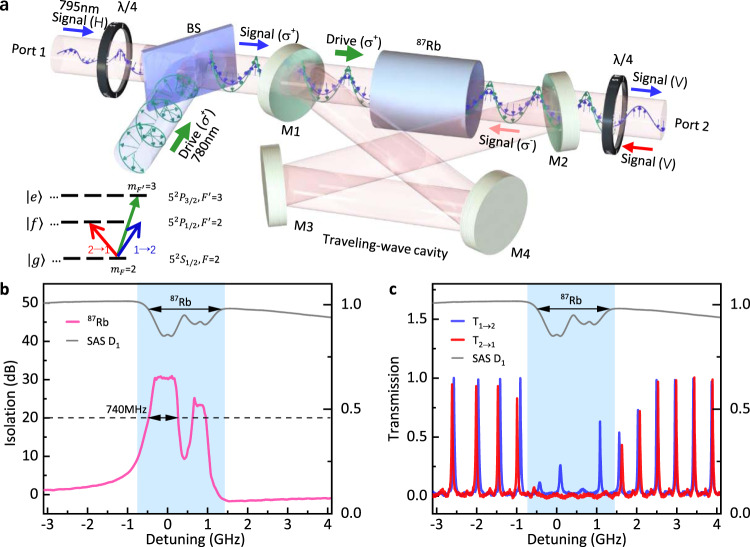
Experimental observation of the magnetic-free non-reciprocity in an atom ensemble. **a** Experimental setup with a traveling-wave cavity, comprising of four mirrors (M1-M4) with a Rb vapor cell inside. The linearly polarized (H: horizontal polarization, V: vertical polarization) input signal from port 1(2) is converted to *σ*^+^(*σ*^−^) polarization by quarter waveplates (*λ*/4). A drive laser in *σ*^+^ polarization is coupled into the cavity through a beam splitter (BS) of port 1. Therefore, the circular birefringence or dichroism leads to non-reciprocal transmission of the whole device system. The inset diagram shows the energy levels of Rb atom, with the arrows representing the drive laser (green), the forward signal laser from port 1 (blue) and the backward signal laser from port 2 (red). **b** The spectrum of broadband isolation by hot atom ensemble due to optically-induced magnetization, based on the setup in **a** without the cavity. A maximum isolation ratio of $30.{3}_{-0.2}^{+0.3}\ {\rm{dB}}$ is observed for ^87^Rb and the corresponding 20 dB-isolation (dashed line) bandwidth is 740 MHz. Here, the drive power (*P*_d_) is about 130 mW while the signal power (*P*_s_) is about 1 μW. **c** Typical transmission spectra for the cavity setup, with *P*_d_ = 45 mW and *P*_s_ = 1 μW. The gray lines is a reference saturated absorption spectrum (SAS) of ^87^Rb D1 line, and the zero frequency detuning corresponds to the transition $|{5}^{2}{S}_{1/2},F=2\rangle \to |{5}^{2}{P}_{1/2},F^{\prime} =1\rangle$. The blue shadow indicates the absorptive region that exhibits circular dichroism, and the region outside the shadow shows mode splitting due to circular birefringence.

We experimentally implement the proposed scheme based on ^87^Rb warm atom vapor, as shown in Fig. 2(a). The energy diagram of the atom is shown in the inset, with $|g\rangle =|{5}^{2}{S}_{1/2},F=2\rangle$ and $|f\rangle =|{5}^{2}{P}_{1/2},F^{\prime} =1,2\rangle$ for the *D*_1_ transitions at ~ 795 nm, and we choose the ancillary energy levels $|e\rangle =|{5}^{2}{P}_{3/2},F^{\prime} =1,2,3\rangle$ corresponding to *D*_2_ transitions at ~780 nm. First of all, the non-reciprocity via OIM is verified by a circularly polarized drive field (~780 nm) on the atoms, with *σ*^+^-polarized forward (port 1 → 2) or *σ*^−^-polarized backward (port 2 → 1) signal probing the vapor cell at ~ 795 nm. Here, the predicted circular dichroism of atomic medium is converted to non-reciprocal transmittance (*T*_1→2_ ≠ *T*_2→1_) of linear polarization introduced by way of a pair of quarter waveplates (Fig. 2(a)). As plotted in Fig. 2(b), we observe a 20 dB isolation with bandwidth of about 740 MHz due to the Doppler broadening from the atom transition linewidth of ~6 MHz. The highest isolation ratio of $30.{3}_{-0.2}^{+0.3}\ {\rm{dB}}$ is achieved without a cavity in a single pass configuration (details in Supplementary Fig. 1). To valid the mechanism for other atomic media, we also demonstrate the cavity-less isolation by the isotope ^85^Rb in a vapor cell of natural abundance Rubidium, demonstrating a 900 MHz bandwidth and a highest isolation ratio of $29.{6}_{-0.6}^{+0.6}\ {\rm{dB}}$. Since the magnetization of the medium could be perturbed by the stray magnetic field perpendicular to the direction of light propagation (*B*_⊥_), the OIM-induced non-reciprocity might be sensitive to magnetic field. However, in our experiments, we found that the isolation is very robust and could be maintained even with *B*_⊥_ = 10 Gauss. Therefore, the OIM provides a very robust photonic non-reciprocity against experimental imperfections, and also relaxes the requirements of drive laser and operating environment for practical applications.

### Absorptive and dispersive non-reciprocity

Since the OIM-induced non-reciprocity strongly depends on the optical depth of the atom medium, which might be a limiting factor for cold atoms or emitters in solids, we investigate the cavity-enhanced non-reciprocity in the following experiments (Fig. 2(a)). Typical spectra of the cavity-enhanced non-reciprocity are shown in Fig. 2(c), where the shadow section corresponds to the absorptive region in Fig. 1(d). Here, the transmission of *T*_2→1_ is greatly suppressed, while peaks with regular free-spectral range are observed in *T*_1→2_ transmission. Since the atoms in the vapor cell fly between the reservoir and cavity mode fields, which induces the relaxation of the OIM, the peaks in the absorption region show reduced transmittance due to the residual populations on the ground states *m*_*F*_ < 2 (*F*_*g*_ = 2). In contrast, in the detuned frequency regions, the system shows resonance shift between forward and backward spectra. These observations confirm the absorptive and dispersive non-reciprocity realized by the OIM in the atomic medium (Fig. 1(d)).

**Fig. 3:**
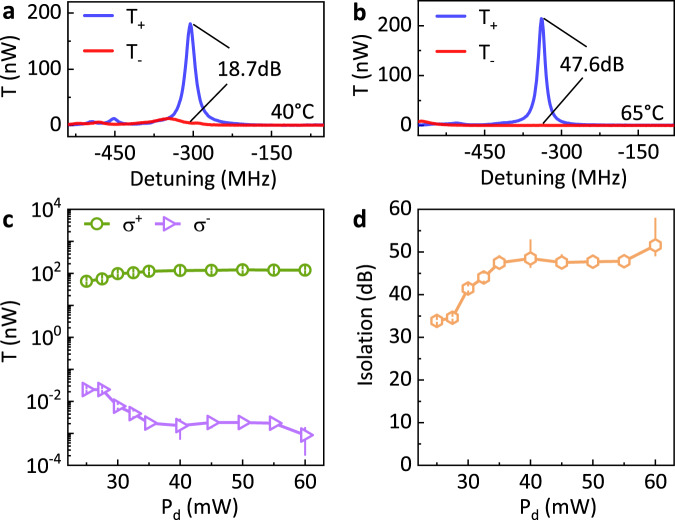
Optical isolation via absorptive non-reciprocity. **a**, **b** Typical transmission spectra for *σ*^+^ and *σ*^−^-polarized signals at different atomic densities, with the temperature of the atomic cell respectively set at 40 ^∘^C and 65 ^∘^C and the corresponding isolation ratios 18.7 dB and 47.6 dB, respectively. Here, the signal and drive power is fixed at *P*_s_ = 50 μW and *P*_d_ = 45 mW, respectively. **c** The on-resonant transmitted signal power vs the drive laser power (*P*_d_). The green and purple spots represent the *σ*^+^ and *σ*^−^-polarized signals, respectively. **d** Extracted isolation ratio at different *P*_d_. The largest isolation ratio $51.{5}_{-2.5}^{+6.5}\ {\rm{dB}}$ is achieved at *P*_d_ = 60 mW. The results in **c** and **d** are measured at 65 ^∘^C with a fixed signal laser power of *P*_s_ = 50 μW, and error bars denote standard deviations.

In an optical traveling-wave cavity, optical modes of orthogonal polarizations are degenerated. As demonstrated above, the OIM could induce the circular birefringence and circular dichroism and thus lifts the degeneracy between *σ*^+^- and *σ*^−^-polarized modes in our system. Then, the non-reciprocity is realized by probing the OIM via quarter waveplates, as the signal transmission for port 2 → 1 with *σ*^−^-polarization is rejected by the cavity. For better calibration of the system performance, we probe the *σ*^−^-polarization from the forward direction by utilizing the symmetry of the system, because all optical elements are the same when measuring both *σ*^+^ and *σ*^−^ polarization forwardly (*T*_+_ and *T*_−_) except that the angle of waveplate is different. Detailed performances of the optical isolation are summarized in Fig. 3. By increasing the temperature of the cell (Fig. 3(a) and (b)), the isolation ratio increases due to the increase of atom density under a fixed drive power *P*_d_ of 45 mW. The optical performance also depends on the drive power. As shown in Fig. 3(c), the *T*_+_ is suppressed at low *P*_d_, because the OIM is weak and the atom ensemble is absorptive for both polarizations. By increasing *P*_d_, the atoms are effectively magnetized and become transparent to the *σ*^+^-polarized signal. Figure 3(d) shows the extracted isolation ratio, which increases with *P*_d_ and reaches a maximum of $51.{5}_{-2.5}^{+6.5}\ {\rm{dB}}$ when *P*_d_ = 60 mW. The linewidth here at 65 ^∘^C is around $26.{5}_{-0.2}^{+0.2}\ {\rm{MHz}}$, primarily determined by the glass-cell-induced loss.

**Fig. 4:**
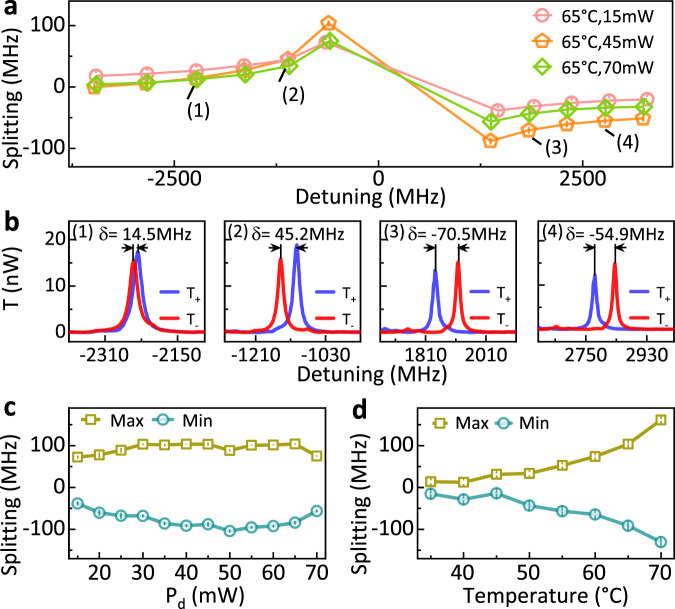
Mode frequency shift due to dispersive non-reciprocity. **a** The resonant frequency shift of two circularly polarized modes at different drive laser power levels (*P*_d_) and fixed cell temperature of 65 ^∘^C. When the frequency of signal is near-resonant to the atoms (blue shadow region in Fig. 2c), the absorptive effect dominates and the resonances for *T*_−_ are suppressed. The dispersive shift can not be distinguished in this regime. **b** The relative mode spectral shifts (*δ*) at four different cavity detunings labeled in **a**. **c** and **d** The extracted maximum and minimum mode frequency shifts against *P*_d_ and different cell temperatures. In all studies, the signal laser power is fixed at *P*_s_ = 20 μW, and error bars denote standard deviations.

Figure 4 presents the results of the dispersive non-reciprocal effects. The resonance shift *δ* = *f*_+_ − *f*_−_ of the *T*_+_ and *T*_−_ spectra at different temperature are shown in Fig. 4(a), where *f*_+(−)_ is the resonant frequency of *T*_+_ (*T*_−_) spectrum. Figure 4(b) further details the transmission spectra corresponding to four detuning frequencies indicated in Fig. 4(a). At 65 ^∘^C and *P*_d_ = 45 mW, we find that the ratio between mode splitting and linewidth *δ*/*γ* reaches 4.55, where *γ* denotes the linewidth of the cavity at *f*_+_. The *δ*/*γ* is a figure-of-merit characterizing OIM for isolator and gyrator to account for non-reciprocal phase shift induced by OIM. Furthermore, we find that the relative frequency shift monotonously increases with the vapor temperature as shown in Fig. 4(d) due to the increase of atom density at elevated temperatures. For the power dependence shown in Fig. 4(c), the frequency shift initially increases and then drops when *P*_d_ approaches 70 mW due to population transfer from atomic ground states to the ancillary energy levels ($\left|f,{m}_{{F}^{\prime}}=3\right\rangle$) at high drive intensities, since the drive is coupling with a cyclic transition ($\left|g,{m}_{F}=2\right\rangle \to \left|f,{m}_{{F}^{\prime}}=3\right\rangle$).

### Noiseless isolation

**Fig. 5:**
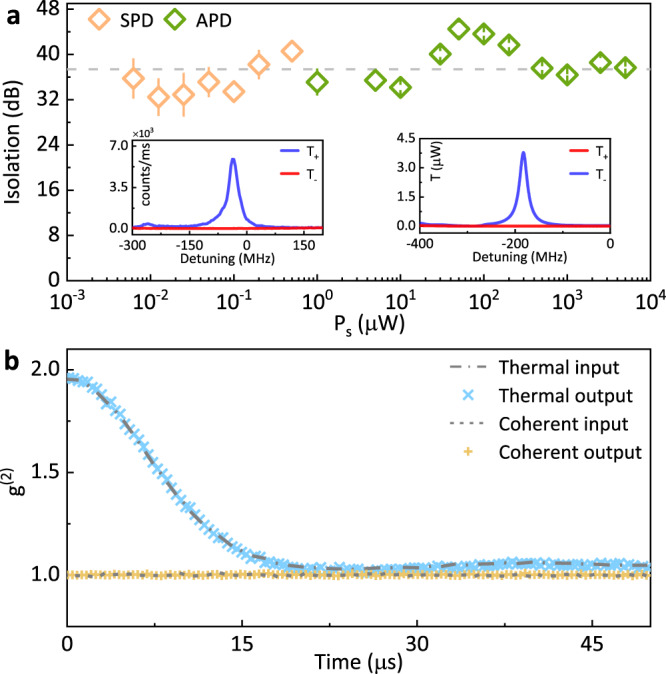
The dynamic range and noiseless property of the all-optical isolation. **a** The measured isolation ratios against input signal power (*P*_s_). Due to the limitation of the system insertion loss and detector’s response, avalanche photodetectors (APDs) are adapted for *P*_s_ ≥ 1 μW (shown as green spots) and a single-photon detector (SPD) is used for *P*_s_ < 1 μW (shown as orange spots). The insets are two typical spectra measured for *P*_s_ = 6.25 nW (left) and 1.2 mW (right), respectively. **b** Measured second-order correlation function (g^(2)^) for signal input to and output from the device. Blue crosses and dashed line are the results for pseudo-thermal source, while yellow crosses and dotted line are the results for coherent source. In all studies, the drive power is set at 50 mW, vapor temperature is 65 ^∘^C, and error bars denote standard deviations.

We lastly demonstrate the inherent noiseless property of our all-optical non-reciprocity via OIM. In the previous demonstration of all-optical non-reciprocity, the strong drive would induce considerable noise by four-wave mixing amplification^18,20,36^ or the thermal noise occupation of low-frequency excitations^12,22^. In contrast, there are no such noise processes in our approach that could excite the *D*_1_ transitions by the drive at room temperature, thus there should be no noise photon generated at signal wavelengths. We verify this characteristic over a wide signal power range varying from nano- to milli-Watt, corresponding to an intracavity photon number in the range of 0.84 − 10^6^. As shown in Fig. 5(a), the isolator shows an average of about 37.4 dB isolation with a signal power dynamic range of about 70 dB. Fluctuations of the performance are attributed to the varied response of the detectors at different input power, such as the saturated gain of avalanche photodetector (APD) and counts saturation of single-photon detector (SPD).

In practical experiments, it is demanding to directly count the noise photons introduced by the isolator when signals transmit through it, because the number of potential noise photons should be weaker compared to the dark counts of the SPD. Instead, we indirectly verify the noiseless isolation by demonstrating the quantum-statistic conservation of the input signal, which is required for the potential applications of magnetic-free non-reciprocity in the quantum domain. As presented in Fig. 5(b), the second-order correlation function ${g}^{(2)}\left(t\right)$ with a delay time *t* is measured for both coherent laser and pseudo-thermal light source (see Method and Supplementary Note 5 for details). By comparing the ${g}^{(2)}\left(t\right)$ curves for input and output signals, we find that its quantum statistic preserves after the isolator. To further quantify the number of noise photons introduced by the isolator, we theoretically analyze the experimental results with the assumption that possible stray thermal and coherent photons are detected by SPD. For a weak probe signal, whose intracavity mean photon number is about 0.84, we derive an equivalent added intracavity noise photon number of *n*_add_ ~ 0.0084 with a confidence level of 99.7% (See Supplementary Note 5 for details), which confirms the noiseless non-reciprocity mechanism aforementioned.

## Discussion

To evaluate the performance of our OIM-based isolator, we compare our devices with other state-of-the-art magnetic-free isolators. In Table 1, the key metrics of the representative experimental works are summarized. Among all these experiments, the quantum noise property has been first characterized in this work. In principle, because the low energy phonons or spin excitations are involved, the optomechanical approach in optical microcavities^13,14^, and also the population trapping or electromagnetically-induced transparency approach in hot atom system^17,32^ suffers from the noises due to the thermal excitations. The isolators based on the Kerr effect^18,31^ in a microcavity are also intrinsically noisy, because the drive-laser-stimulated parametric process could amplify the signal and thereby produce noise photons. Although the acousto-optical approach for the isolator^28–30^ should be free of the thermal and parametric noises, the signal passing the isolator is not frequency-preserving, and suffers from the extra phase modulation as well as the cross-talk error with other integrated photonic components due to the strong on-chip acoustic drive. Therefore, their noise performances require further experimental investigations. In terms of the isolation ratio, the OIM-induced non-reciprocity in a cavity outperforms all other devices, despite of a relatively low bandwidth and high insertion loss. Our theoretical analysis (Supplementary Note 4) predicts a 1 dB insertion loss and 100 MHz forward transmission bandwidth by optimizing the mirror reflectivities. For practical applications, the OIM-induced non-reciprocity in free space is attractive for its overall performances including its noiseless property, large bandwidth approaching GHz, and low insertion loss ~ 1 dB. It is anticipated that the bandwidth could be further improved by introducing other isotopes (for example, the results of combined ^85^Rb and ^87^Rb are provided in Supplementary Fig. 1) and the collision effect of hot atoms^32^.

**Table 1:** Comparison with other representative experimental demonstrations of the magnetic-free optical isolator. The table compares our devices with other state-of-the-art devices, in terms of platform, principle, max isolation ratio, 3 dB forward transmission bandwidth, 20 dB isolation bandwidth, and insertion loss. PIC: photonic integrated circuits, EIT: electromagnetically-induced transparency, OIM: optically-induced magnetization. The symbol ^*^ indicates that the value is derived based on the reported experimental results.

Reference	Platform	Principle	Max isolation ratio	3 dB Forward Transmission Bandwidth	20 dB isolation bandwidth	Insertion Loss
^14^	Microcavity	Optomechanics	12.8 dB	22 kHz	0	2.1 dB
^13^	Microcavity	Optomechanics	10 dB	88 kHz	0	~10 dB
^18^	Microcavity	Kerr effect	~18 dB	3 MHz	0	~4 dB
^31^	Microcavity	Kerr effect	24 dB	1.3 MHz^*^	1.3 MHz^*^	7 dB
^28^	Microcavity	Acousto-optic	~15 dB	1.14 GHz	0	7.7 dB
^29^	Waveguide	Acousto-optic	38 dB	125 GHz	~100 GHz^*^	18.8 dB
^30^	Waveguide	Acousto-optic	16 dB	100 GHz	0	8.7 dB
^32^	Hot Atoms (Free-space)	Population Trapping	40 dB	no limitation	1.2 GHz	~1 dB
^17^	Hot Atoms (Cavity)	EIT	14.5 dB^*^	~4 MHz	0	2.7 dB^*^
This Work	Hot Atoms (Free-space)	OIM	30.3 dB	no limitation	740 MHz	1.5 dB
This Work	Hot Atoms (Cavity)	OIM	51.5 dB	16.9 MHz	>100 MHz	~20 dB

The potential extension of the OIM to integrated photonic devices on a chip is particularly appealing. The demonstrated noiseless non-reciprocity only requires an atomic ensemble with the degenerate Zeeman energy levels, thus the mechanism is applicable to many atoms or atom-like emitters, including hot and cold atoms, molecules, as well as emitters in solids (such as NV centers and rare-earth atoms). There are several potential approaches to integrate these emitters with the waveguides, including the fabrication of hot-atom-cladding waveguide by bonding atomic vapor cell to photonic integrated circuits^37–40^, trapping cold atoms in the evanescent field of the waveguide^41,42^, transferring molecules and nanocrystals on the surface of waveguides^43,44^, as well as implanting and doping ions in the waveguide or the cladding layer^45,46^. However, the optical waveguide modes on photonic chips are distinct from the laser beam in free space, and the configuration in Fig. 1 could not be directly applied because the conventional circularly-polarized optical field is only applicable for integrated photonic waveguides with a circular cross-section. Luckily, there is a mechanism of optical spin-orbit coupling in photonic waveguides^33,47,48^, which enables the realization of the OIM by harnessing transverse circularly-polarized optical fields that are perpendicular to the light propagation direction^40^. For instance, the field distributions of co-propagating drive and signal lasers in fundamental mode are almost the same, thus each individual atom couples with *σ*^+^-polarized (or *σ*^−^-polarized, the polarization is spatially dependent^33,47,48^) field of both drive and signal lights simultaneously, then realizing the circular birefringence and dichroism in Fig. 1(a). As a promising goal following our demonstration in this work, the OIM in integrated devices have many potential advantages, including smaller footprint, reduced pump power due to the enhanced light-atom interaction by the waveguide, and broader bandwidth due to the stronger spectral broadening.

In conclusion, the concept of the optically-induced magnetization and its sequential applications in high-performance optical isolation are demonstrated for the first time. Comparing to previously demonstrated all-optical non-reciprocity based on the coherent nonlinear processes, the optically-induced atomic magnetization is an incoherent process, which is very robust against experimental imperfections, such as the drive frequency and amplitude fluctuations, inhomogeneous transition frequency broadening, imperfect circular polarization, and beam alignment, as well as stray magnetic fields. The mechanism is general and could be extended to various wavelength bands, e.g., UV or mid-IR wavelengths where commercial products have limited performance so far, and also to different device platforms, e.g., integrated photonic devices as well as phononic devices by utilizing their interaction with electron spins in solids^49^. Aside from the potential applications, our work promises new physics by taking the degenerate ground Zeeman energy levels into cavity quantum electrodynamics (QED)^50^ and waveguide QED^51^, where interesting non-reciprocal phenomena arise, such as non-reciprocal multi-stability, quantum frequency conversion, photon storage, and lasing.

## Methods

### Experimental setup

The photonic non-reciprocity is validated by optically-induced magnetization of Rubidium (^87^Rb) atoms, in both scenarios with and without an optical traveling-wave cavity. A 75 mm-long vapor cell is filled with pure ^87^Rb, heated by a soft film heater and stabilized by a temperature controller (Thorlabs, TC200). Two external cavity diode laser sources (Toptica, DL pro 780 nm and 795 nm) are used as the drive and signal lasers, corresponding to the *D*_2_ and *D*_1_ transitions of the Rb atoms. Both lasers are coupled to the system through fibers, and match the fundamental modes of the cavity by two convex lenses with the same focal length at 25 mm. A repump laser, with frequency different from the drive laser by about 6.8 GHz and power around 10 mW, is combined with drive laser and applied to the system for achieving a better performance of non-reciprocity. Note that the repump laser is not necessary for demonstrating the non-reciprocity mechanism studied in this work, while it would effectively increase the optical depth of the atomic medium at a moderate temperature.

In the case without a cavity, the atom vapor cell is directly driven by laser from forward direction, and a signal laser probes the system from either forward (port 1 → 2) or backward (port 2 → 1) direction. In the case with a cavity, we build a traveling-wave cavity and place the atom vapor cell inside it for an enhanced optically-induced magnetization effect. The cavity composes of four mirrors. The input and output ports of the cavity are flat mirrors, with the reflectivities of 91.8% and 99.6%, respectively, and the other two mirrors are concave mirrors with the same curvature radius of 100 mm and a high reflectivity over 99.9%.

### Characterization of non-reciprocity

Both signal and drive lasers are circularly polarized, with the polarization being adjustable by quarter waveplates. On both sides of the cell or cavity-cell system, beam splitters are utilized to combine the drive and signal lasers. Polarized beam splitter are used to separate the drive and probe lasers when their polarizations are orthogonal. In addition, interference band-pass filters (Union Optic, ITF9125-795nm) are used for rejecting the drive lasers with very extinction ratio, because the wavelength difference between the two lasers is about 15 nm. When measuring the signal power dynamic range of the device, we use avalanche photodetectors (Thorlabs APD410A, and also Newport 2151), and single-photon detectors (Excelitas, SPCM-800-24-FC) at certain power ranges. The transmission spectra of signal are detected and recorded by avalanche detectors, with a probe power range 1–100 μW. When characterizing the isolation ratio by APD, it is very challenging to measure the very weak signal that is isolated by the device. Therefore, we modulate the signal light (1.5 kHz) and measure the weak signal precisely by a lock-in amplifier (Zurich Instruments, MFLI 500 kHz).

### The quantum statistic measurement

To characterize the noise properties of the device, the quantum statistics of transmitted signal from both coherent and pseudo-thermal sources are tested experimentally. The coherent source is generated by attenuating the signal laser. The pseudo-thermal source is built by focusing a laser on the rough surface of a rotating ground glass disk (Thorlabs, DG20-1500). The second-order photon correlation function (second-order degree of coherence) of the pseudo-thermal source shows a bunching quantum statistics, i.e., the typical ${g}^{(2)}\left(0\right)$ is about 2, in contrast to ${g}^{(2)}\left(t\right)=1$ of a coherent source. In our system, we measure the second-order photon correlation function by two single-photon detectors (Excelitas, SPCM-800-24-FC) and a time-to-digital converter (quTAG, standard 4 channels). Both coherent and pseudo-thermal sources are characterized before they are coupled into the cavity, and the results are served as a reference for that of the photons transmitted through the cavity. During the quantum statistic measurements, the frequency of the signal is fixed for the highest isolation ratio. The second-order photon correlation function of the output matches the input very well, which provides the evidence that the device is noiseless and could be useful in future quantum applications.

## Supplementary information

Supplementary Information

## Data Availability

The data that support the findings of this study are available from the corresponding authors upon reasonable request.

## Code availability

The code used for simulations is available from the corresponding authors upon reasonable request.
