# Supplementary material for: Noiseless photonic non-reciprocity via optically-induced magnetization
Source: Nat Commun. 2021 Apr 22;12:2389. doi: 10.1038/s41467-021-22597-z (PMC8062452; doi:10.1038/s41467-021-22597-z)
Supplement: Supplementary file 1 — Supplementary Information [file 41467_2021_22597_MOESM1_ESM.pdf]

Supplementary Material for "Noiseless photonic non-reciprocity via optically-induced magnetization"

Xin-Xin Hu,[1,3,*] Zhu-Bo Wang,[1,3,*] Pengfei Zhang,[2,4,†] Guang-Jie Chen,[1,3] Yan-Lei Zhang,[1,3] Gang Li,[2,4] Xu-Bo Zou,[1,3] Tiancai Zhang,[2,4] Hong X. Tang,[5] Chun-Hua Dong,[1,3,‡] Guang-Can Guo,[1,3] and Chang-Ling Zou[1,3,2,§]

[1]*CAS Key Laboratory of Quantum Information, University of Science and Technology of China, Hefei 230026, P. R. China.*
[2]*State Key Laboratory of Quantum Optics and Quantum Optics Devices, and Institute of Opto-Electronics, Shanxi University, Taiyuan 030006, China*
[3]*CAS Center For Excellence in Quantum Information and Quantum Physics, University of Science and Technology of China, Hefei, Anhui 230026, P. R. China.*
[4]*Collaborative Innovation Center of Extreme Optics, Shanxi University, Taiyuan 030006, China*
[5]*Deparment of Electric Engineering, Yale University, New Haven, CT 06511, USA*

(Dated: March 9, 2021)

SUPPLEMENTARY NOTE 1 - EXPERIMENTAL SETUP

A. Setup for free space non-reciprocity

The experimental setup without a cavity is shown in Supplementary Fig. 1(a). A 75 mm glass vapor cell filling with pure ^{87}Rb atoms is wrapped by a soft heater, and its temperature is stabilized by a temperature controller (Thorlabs, TC200) with a uncertainty less than 0.1 °C. Similar setup for the cavity and vapor cell system can be found in Ref. [1]. A kit of polarization components including a half waveplate (HWP), a polarization beamsplitter (PBS) and a quarter waveplate (QWP) is employed to control the polarization of the drive laser to be nearly-perfect σ^+-polarization. Shown as green paths in Supplementary Fig. 1(a), the drive laser resonant with the D_2transition $|5^2S_{1/2}, F=2\rangle \rightarrow |5^2P_{3/2}, F'=3\rangle$ is injected into the glass vapor cell forwardly from Port 1 → Port 2. The circularly polarized laser drives the atoms and initializes atoms to the Zeeman sub-energy level at $|5^2S_{1/2}, F=2, m_F=+2\rangle$, which could be treated as magnetized optical medium. Therefore, the laser could realize the optically-induced magnetization (OIM) along the laser propagation direction. However, due to inhomogeneous broadening of the atom ensemble induced by Doppler effect, the drive laser could also induce the transition from ground states to $|5^2P_{3/2}, F'=2\rangle$, and then sequentially the excited atoms could decay to the dark state $|5^2S_{1/2}, F=1\rangle$ via the spontaneous emission. After that, these atoms are absent in the interaction with signal and drive lasers, thus the drive laser could effectively reduce the density of atoms that participating in the light-matter interaction. To enhance the effective atom density at a low temperature, an additional repump laser resonant with the transition $|5^2S_{1/2}, F=1\rangle \rightarrow |5^2P_{3/2}, F'=2\rangle$ is combined with the drive laser to repump the atoms in the dark state back to the active states, as shown in Supplementary Fig. 1(b). Both circularly and linearly polarized repump lasers are useful and available in our experiments, but we choose the polarization of the repump to be the same as the drive laser to simplify the experimental apparatus. Note that we fix the power ratio of drive and repump lasers at about 3 : 1.

The weak signal laser scanning across the D_1transition of ^{87}Rb is injected into the system through two ports (Port 1 and Port 2), as shown in Supplementary Fig. 1(a). From Port 1, the horizontally polarized signal from a polarization-maintaining (PM) fiber is collimated by collimator C_1 and converted to σ^+ polarization by a polarization kit. A beamsplitter (BS) is employed to combine the drive and signal lasers. After transmitting through the glass vapor cell, both the drive and signal lasers are altered to vertical polarization, shown as the blue arrows in Supplementary Fig. 1(a). Due to the atomic population transfer under OIM, the σ^+ dipole transition of ^{87}Rb is forbidden for signal light and the transmitted signal can be detected subsequently by a photodetector (D_2), before which the drive laser is rejected by cascaded interference band-pass filters (Union Optic, ITF9125-795nm). In contrast, the signal laser injected to Port 2 (shown as red arrows in Supplementary Fig. 1(a)) is vertically polarized and can be converted to σ^- polarization by the same polarization kit without changing the angle of waveplates. Since the σ^- dipole transition is allowed, the signal could interact with the atomic medium and experience absorption loss, and eventually the power of the transmitted signal detected by photodetector D_1 is extremely weak signal.

For the free space case, the isolation ratio I (dB) is defined as

$$I = 10\log\frac{T_{1\rightarrow2}}{T_{2\rightarrow1}}, \tag{1}$$

where $T_{1\rightarrow2}$ is the transmission laser power from Port 1 → Port 2 and $T_{2\rightarrow1}$ corresponds to that of the opposite direction. In our experiments, the weak transmitted signal $T_{2\rightarrow1}$ is detected with the assistance of a lock-in amplifier (Zurich Instruments, MFLI 500kHz) for an enhanced signal-to-noise ratio (SNR). In the free space experiments, the input signal power is about 1 μW while the drive and repump powers are around 100 mW and 30 mW, respectively.

B. Setup and additional results for cavity-enhanced non-reciprocity

Supplementary Fig. 2(a) depicts the experimental setup for realizing the cavity-enhanced non-reciprocity. We build a traveling-wave Fabry-Perot (FP) cavity by four mirrors, which composes of two mirrors with reflectivities of 91.8 % (M1) and 99.6 % (M2) respectively, and two mirrors with the reflectivity of 99.9 % (M3, M4). The measured free spectral range (FSR) and linewidth of the bare cavity are 515 MHz and 7.3 MHz, respectively, which indicate a cavity length of ∼ 582 mm and a finesse of ∼ 70. After placing a glass vapor cell into the cavity, the finesse reduces to ∼ 20 due to the absorption and scattering losses by the atomic medium and glass windows. The detailed calibration of the cavity parameters is provided in the Section B. A piezoelectric transducer (PZT) is mounted on M4 to control and stabilize the cavity length, so that we can lock it to the drive laser by a feedback loop. Two external cavity diode laser sources

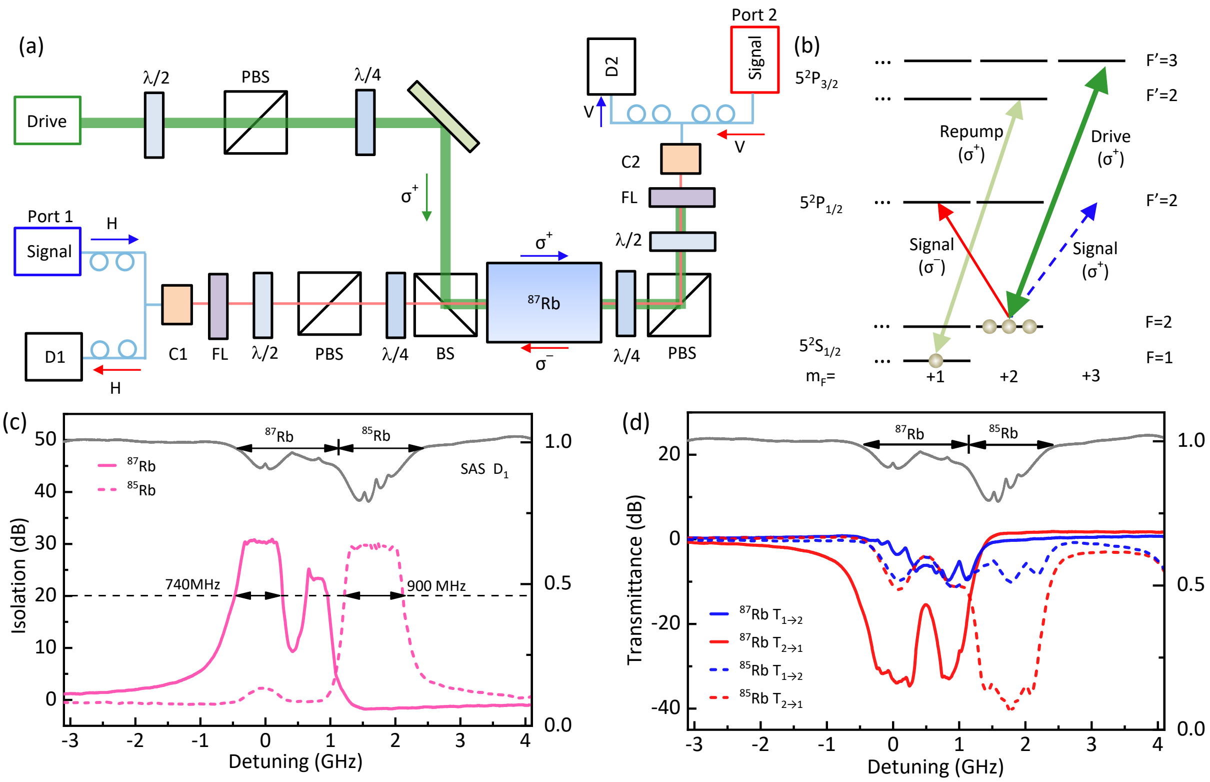

Supplementary Figure 1. **Setup for realizing the free space non-reciprocity.** (a) The experimental setup for studying the OIM-based non-reciprocity without cavity. The pink lines denote the optical paths of the signal laser while the green lines denote the paths of the drive laser. D1,D2: photodetectors; $C_{1,2}$, collimators; $\lambda/2$: half waveplate (HWP); $\lambda/4$: quarter waveplate (QWP); BS: beamsplitter; PBS: polarization beamsplitter; FL: cascaded filters. (b) The detailed energy level diagram of the atoms. The green arrow resonant with the transition $|5^2S_{1/2}, F=2\rangle \to |5^2P_{3/2}, F'=3\rangle$ indicates the drive laser, while the jade-green arrow corresponds to the repump laser, which is resonant with the transition $|5^2S_{1/2}, F=1\rangle \to |5^2P_{3/2}, F'=2\rangle$. By the drive and repump lasers, the population of atom ensemble could be polarized to the Zeeman sub-energy level $|5^2S_{1/2}, F=2, m_F=+2\rangle$, therefore only the σ^- dipole transition is available (red arrow) while the σ^+ dipole transition is forbidden (blue dashed arrow), which eventually breaks the time-reversal symmetry of the signal. (c) The spectrum of broadband isolation by hot atom ensemble due to optically-induced magnetization, based on the setup in (a) without the cavity. A maximum isolation ratio of $30.3^{+0.3}_{-0.2}$ dB and $29.6^{+0.6}_{-0.6}$ dB is observed for ^{87}Rb and ^{85}Rb, respectively. The dash line indicates the 20 dB isolation ratio, and the corresponding 20 dB-isolation bandwidth for ^{87}Rb and ^{85}Rb are 740 MHz and 900 MHz, respectively. (d) The corresponding transmission spectra for ^{87}Rb and ^{85}Rb hot atom ensemble, respectively.

provide the drive and signal light. The drive laser is locked to the transition $|5^2S_{1/2}, F=2\rangle \to |5^2P_{3/2}, F'=3\rangle$ by the saturated absorption spectrum (SAS) and the signal laser scans across the D_1transition of ^{87}Rb. The two lasers are coupled to the cavity through a selected port, and the beam profiles are adjusted via two lenses with the same focal length of 25 mm to match the fundamental mode of the FP cavity, as shown in Supplementary Fig. 2(a). For a better calibration of the system isolation ratio, we measure the σ^+ and σ^- spectra from the same direction instead of measuring the spectra from two ports as shown in Supplementary Fig. 2(b). This is because that for weak signal coupling with the cavity, the transmission should be the same for both forward and backward direction (see Sect. B for details). However, the calibration of the forward and backward optical paths is difficult due to different optical components are employed in different paths. By adjusting the angle of waveplate Q_1 in Supplementary Fig. 2(a), both σ^+ and σ^- signal transmission can be detected from Port 1 $\to$ Port 2 due to the symmetry of the circularly polarized laser. Therefore, the isolation ratio I for the case with a cavity case can be written as

$$I = 10\log\frac{T_+}{T_-}, \tag{2}$$

where $T_{+(-)}$ corresponds to the $\sigma^{+(-)}$-polarized input signal laser.

Similar to the free space case, drive and repump lasers of the cavity-enhanced non-reciprocity are near-resonant with the two transitions of ^{87}Rb D_2 line, and atoms can be polarized to the Zeeman sub-energy level $5^2S_{1/2}$, $F=$

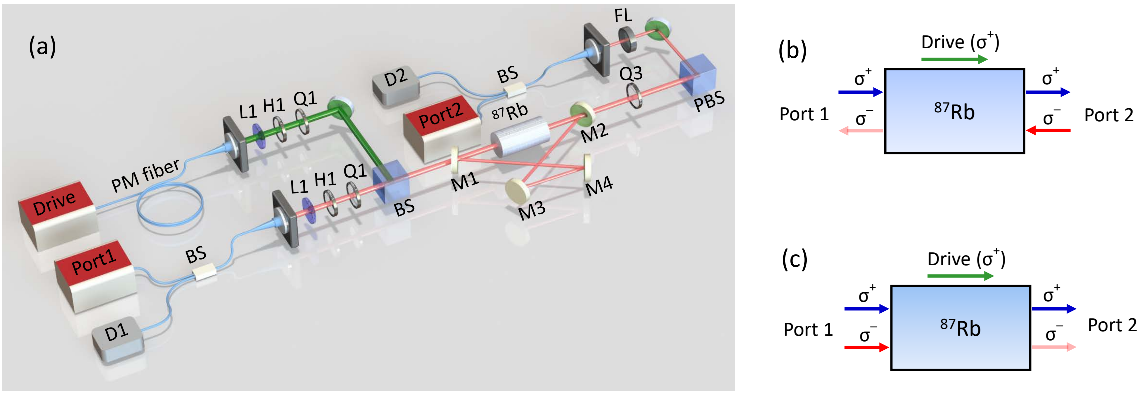

Supplementary Figure 2. **Detailed experimental setup for cavity-enhanced non-reciprocity.** (a) The experiment setup of the system with a traveling-wave cavity. Similar to the free space setup, the pink lines show the free space optical paths of the signal laser while the green lines indicate the drive laser. The drive and signal lasers are coupled to the cavity via PM fibers. L1,L2: the mode-matching lenses with the focal length of 25mm; H1,H2: half waveplates; Q1,Q2,Q3: quarter waveplates; BS: beamsplitter; PBS: polarization beamsplitter; M1, M2, M3, M4: the four mirrors of the cavity; D1,D2: photodetectors. Corresponding to the different signal power, photodetectors can be replaced by photodiodes, avalanche diodes and single photon detectors, respectively. (b) and (c) indicate the schemes of measurement setup. Instead of the scheme (b), we choose scheme (c) in which we measure both the σ^+ and σ^- spectra from the same direction by changing the angle of quarter waveplates.

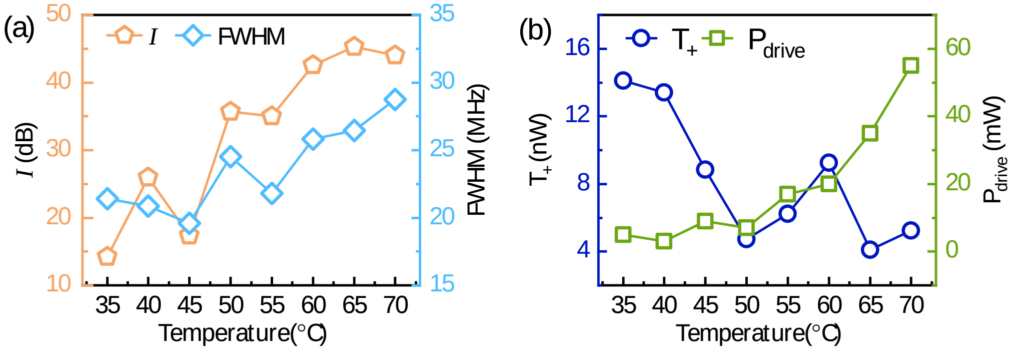

Supplementary Figure 3. **The additional experimental results on the isolation performance against the vapor cell temperature.** (a) The maximum isolation ratio I (orange) and the isolation linewidth (blue) at different temperatures. (b) The transmitted signal power T_+ (blue) and the corresponding drive laser power P_{drive} (green) for obtaining the isolation ratios I in (a).

2, $m_F = +2$ under OIM. To study the dependence of I on the drive power, we vary the drive laser power from 15 mW to 70 mW, with a step of 5 mW. The ratio between drive and repump laser powers is fixed at $5:1$, while the signal laser power is 50 μW. The results are summarized in Fig. 3 in the main text, where the transmitted signal power $T_{+(-)}$ is detected with a lock-in amplifier.

We also characterized the dependence of cavity-enhanced isolation performance on the temperature. The isolation ratio I (orange spots) and linewidth (blue spots) against temperature are summarized in Supplementary Fig. 3(a). Here, the linewidth is defined as the full-width-at-half-maximum (FWHM) for the cavity-allowed transmitted signal (σ^+-polarized). When temperature increases, the isolation ratio I defined by Supplementary Eq. (3) increases because the transmission of σ^--polarized signal laser could be greatly reduced by the atomic absorption. The maximum isolation ratio $I = 45.3$ dB is obtained when the vapor cell temperature approaches 65 °C, hence the results shown in Fig. 3 in the main text are collected under this condition. In Supplementary Fig. 3, the trends of increasing I and FWHM for a increasing temperature imply the enhanced isolation performance due to larger optical depth and larger atom absorption for higher atom density in the vapor cell. Note that the results are measured without the assistance of the lock-in amplifier, thus the SNR is restricted for high isolation ratios, which leads to the saturation of I at high temperatures.

In the spectra in main text, we could find that the transmission of signal could still be suppressed even when it

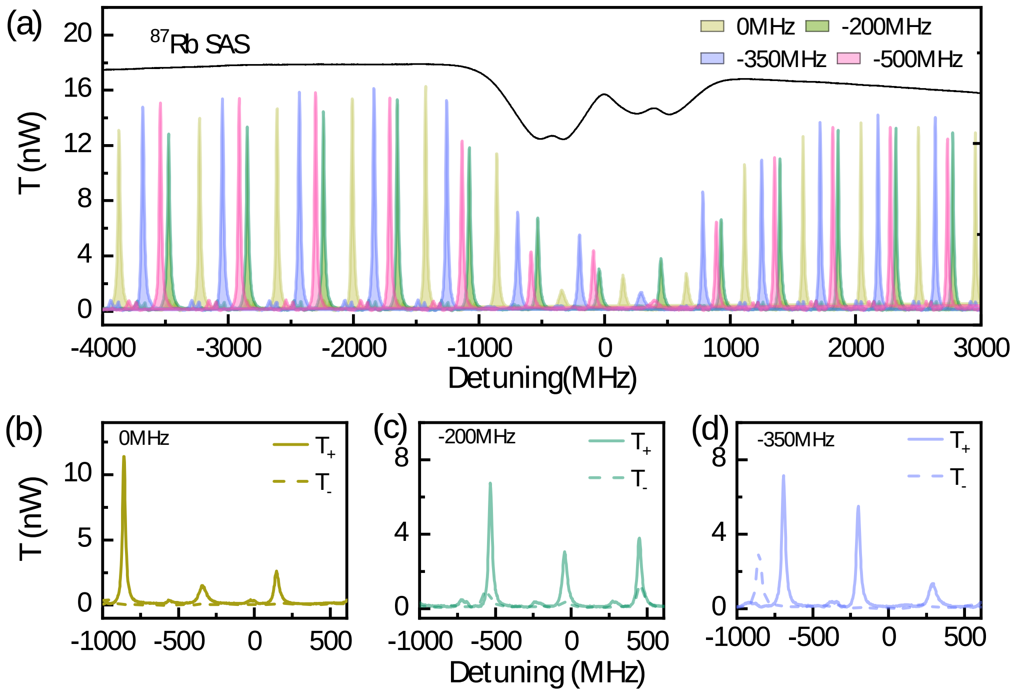

Supplementary Figure 4. **The tuning range of the cavity-based isolator.** (a) The performance of tunable cavity resonance for different cavity length, which is controlled by selecting different drive frequencies. The black line above indicates the SAS of D_1 transition of ^{87}Rb as a comparison. The color lines correspond to the T_+ spectra with the drive laser detuning at 0 MHz, −200 MHz, −350 MHz and −500 MHz, respectively. (b)-(d) Detailed spectra with the drive detuning at 0 MHz (yellow), −200 MHz (green), and −350 MHz (purple). The solid and dashed lines represent T_+ and T_- spectra respectively.

has the same polarization as the drive. The reason is that due to experimental imperfections, there are some residual atoms populated in other Zeeman sub-energy levels, which can be excited by the σ^+-polarized signal and thus leads to absorption of the signal. The imperfections are mainly contributed from three aspects: (1) The imperfection of the drive laser polarization. (2) The limited power of the drive laser. (3) The atoms fly in the vapor cell, and thus induce an effective relaxation of the ground spin state. By increasing the optical depth, the residual atomic population also increases, and therefore T_+ reduces with increasing temperature, as shown by the blue spots in Supplementary Fig. 3(b). The green spots in Supplementary Fig. 3(b) indicate the drive laser power (P_{drive}) which obtains each isolation ratio I in Supplementary Fig. 3(a). All the results prove that the increase of cell temperature can improve the isolator ratio, but cause considerable absorption loss and higher power requirement.

In free space, due to the Doppler effect, we can observe an broad bandwidth of non-reciprocity of ~ 400 MHz by of a room temperature atoms vapor cell. Although the bandwidth of the cavity-based isolation is limited by the mode linewidth, the cavity mode frequencies are tunable by controlling the cavity length. Therefore, we have also investigated the tuning range of the cavity-based isolation, as shown in Supplementary Fig. 4. Since the cavity is locked to the drive laser by a feedback loop, we can demonstrate a tunable cavity-based isolator by adjusting the drive laser frequency, which could easily achieve a ~ 500 MHz tuning range by a double-pass acoustic-optic modulator (AOM) frequency shifter and changing the drive laser locking frequency. Supplementary Fig. 4(a) shows the typical signal transmission spectra with the detuning of drive frequency fixed at a relative detuning of 0 MHz, −200 MHz, −350 MHz and −500 MHz, respectively, and T_+ spectra are measured under different drive frequencies. Here, 0 MHz corresponds to the frequency resonant with the transition $5^2S_{1/2}$, $F = 2 \rightarrow 5^2P_{3/2}$, $F' = 3$, and the black line represents the frequency reference of D_1 transition. To clarify the isolation effect under different drive laser frequency, the detailed transmitted $T_{+(-)}$ spectra are indicated in Supplementary Fig. 4(b)-(d), where $T_{+(-)}$ corresponds to the signal laser in $\sigma^{+(-)}$ polarization. The drive laser frequencies are fixed at the dutuning of 0 MHz, −200 MHz and −350 MHz, respectively. Note that the drive laser frequency can be tuned continuously by changing the RF drives on the AOM, thus we can achieve an exquisite frequency tuning of the isolator.

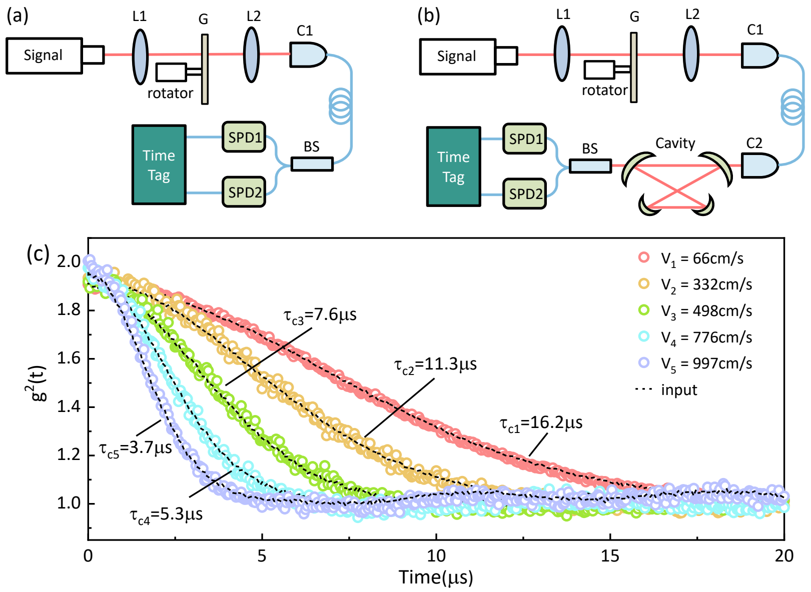

Supplementary Figure 5. **Quantum statistics measurement.** (a) The setup of the pseudo-thermal source and the measurement of $g^{(2)}(\tau)$ curve in free space. (b) The setup of the pseudo-thermal source and the measurement of $g^{(2)}(\tau)$ curve with the cavity-based isolator. (c) The $g^{(2)}(\tau)$ curves of pseudo-thermal signals measured by the apparatus in (a) and (b) with various rotating speeds. The color spots correspond to the signals transmitted through the cavity-based isolator, while the black dashed lines represent the $g^{(2)}(\tau)$ of input signals. The coherent time shown in the figure are fitted by Supplementary Eq. (3). The rotating velocity indicates the linear velocity at the rim.

SUPPLEMENTARY NOTE 2 - THE CHARACTERIZATION OF QUANTUM STATISTICS

To demonstrate the noiseless property of the OIM-based non-reciprocity, we characterize the quantum statistics, i.e. second-order correlation function ($g^{(2)}(\tau)$, with τ is the delay time), of the weak signal transmitted through the device. Since the concept of OIM is intrinsically noiseless, the photon statistics of signal field should be conserved. For instance, the second-order correlation function of an attenuated coherent signal laser is measured for both input and output of the cavity-based device, shown as the yellow crosses and gray dotted line in Fig. 5(b) of the main text. The agreement between input and output curves proves the noiseless properties of the device, but the noise of residual drive laser is rarely concerned. Therefore, a pseudo-thermal input field is also tested as a further evidence of noiseless non-reciprocity.

Apparatus of pseudo-thermal sources are shown in Figs. 5(a) and (b). A disk ground glass (G) mounted on a rotator is employed to generate the pseudo-thermal source. A detailed description of the setup for the pseudo-thermal source can be found in Ref. [2]. When the input signal transmits the rotating G, both temporal and spatial coherence of the signal field can be reduced, in which the coherent time is determined by the rotating speed and spot size focused on the surface of G. The second-order correlation function of Gaussian chaotic light can describe the statistics of pseudo-thermal source, which can be written as

$$g^{(2)}(\tau) = 1 + \exp\left[-\pi\left(\tau/\tau_c\right)^2\right], \tag{3}$$

where τ_c corresponds to the coherent time of the thermal source. The $g^{(2)}(\tau)$ curve of input signal field is measured by the setup shown in Supplementary Fig. 5(a), where two convex lenses with a focal length at 75 mm are used to focus the collimated beam onto the surface of the rotating G. The pseudo-thermal light is separated equally into the two arms of a fiber BS with a splitting ratio of 50:50, and detected by two individual single photon detectors (SPDs, Excelitas SPCM-800-24-FC). The clicks of single photons are collected and summarized by a time-to-digital converter (quTAG, standard 4 channels), and shown as the blue crosses and gray dashed line in Fig. 5 of the main text. The pseudo-thermal light generated by the rotating G is sent into the cavity-based isolator, and its $g^{(2)}(\tau)$ is measured by the setup shown in Supplementary Fig. 5(b). To reduce the impact of background photons from the drive laser, cascaded filters are placed after the cavity.

For further investigation on the quantum statistics, the relation between the coherent time of the pseudo-thermal

light and the rotating speed is measured, and the experimental results are summarized in Supplementary Fig. 5(c). The red, orange, green, blue and purple dots represent the rotating speeds of 66 cm/s, 332 cm/s, 498 cm/s, 776 cm/s and 997 cm/s, respectively. The coherent times labeled in the Supplementary Fig. 5 are fitted by Supplementary Eq. (3), where the results indicate that the increase of rotating speed will reduce the temporal and spacial coherence. It is worth to note that the numerical value of $g^{(2)}(0)$ rise with the rotating speed. It could be explained that the generated pseudo-thermal light is a mixture of a thermal field and a coherent field, where the mixing ratio is determined by the rotating speed. Therefore, the high rotating speed causes a random phase of the input beam due to the moving particles on the glass surface, which reduces the coherence of input signal. For a concrete analysis about the noise introduced by the cavity-based isolator, see Sect. B.

SUPPLEMENTARY NOTE 3 - THEORETICAL ANALYSIS OF HOT ATOM ENSEMBLE

The Hamiltonian for atom-light interaction in the free space system shown in Supplementary Fig. 1(a) can be written as ($\hbar = 1$):

$$\begin{aligned} H = \sum_{j=1}^{N}\sum_{m_F} \Big\{ & \omega_g \left|g, m_F\right\rangle \left\langle g, m_F\right|_j + \omega_f \left|f, m_F\right\rangle \left\langle f, m_F\right|_j + \omega_e \left|e, m_F\right\rangle \left\langle e, m_F\right|_j \\ & + \Omega_d^+ \left(\left|e, m_F + 1\right\rangle \left\langle g, m_F\right|_j e^{-i\omega_d t} + h.c. \right) + \Omega_d^- \left(\left|e, m_F - 1\right\rangle \left\langle g, m_F\right|_j e^{-i\omega_d t} + h.c. \right) \\ & + \varepsilon_s^+ \left(\left|f, m_F + 1\right\rangle \left\langle g, m_F\right|_j e^{-i\omega_s t} + h.c. \right) + \varepsilon_s^- \left(\left|f, m_F - 1\right\rangle \left\langle g, m_F\right|_j e^{-i\omega_s t} + h.c. \right) \Big\}, \end{aligned} \quad (4)$$

where Ω_d (ε_s^+) is the drive (signal) strength with the frequency ω_d (ω_s) and the symbol $+(-)$ means the σ^+ (σ^-)-polarized laser. Here m_F denotes the Zeeman state, where the range of values is $-F_g, -F_g + 1, ..., F_g$ for the state $|g\rangle$, and $F_{f(e)}$ for the state $|f\ (e)\rangle$. Considering the decay of the atom, the dynamics of the system is governed by the Master equation [3], which reads

$$\frac{d\rho}{dt} = -i\left[H, \rho\right] + \sum_{j=1}^{N}\sum_{m_F, m_f'} \left[\gamma_{gf}^{m_F m_F'} \mathcal{L}\left(\left|g, m_F\right\rangle \left\langle f, m_F'\right|_j \right) + \gamma_{ge}^{m_F m_F'} \mathcal{L}\left(\left|g, m_F\right\rangle \left\langle e, m_F'\right|_j \right) \right], \quad (5)$$

where $\gamma_{gf}^{m_F m_F'}$ is the decay rate and the Lindblad super-operator $\mathcal{L}(o) = o\rho o^\dagger - \rho o^\dagger o/2 - o^\dagger o \rho/2$.

Here, we have the energy level $F_e \geq F_g$, thus the σ^+-polarized drive laser Ω_d^+ could change the population of all atoms to $|g, F_g\rangle$. For $F_f \leq F_g$, the transition for the signal ε_s^+ is forbidden, which means the optical susceptibility $\chi_{gf}^+ = 0$. Therefore, the atomic medium is transparent to the signal laser. In contrast, the transition for the signal ε_s^- is allowed. For the single-atom model, we obtain the optical susceptibility

$$\chi_{gf}^- \propto \frac{i}{i\Delta_s + \frac{\gamma_{gf}}{2}}, \quad (6)$$

where $\Delta_s = \omega_f - \omega_g - \omega_s$. The absorption and dispersion for the input signal $\varepsilon_s^\pm$ correspond to $\mathrm{Im}\left(\chi_{gf}^\pm\right)$ and $\mathrm{Re}\left(\chi_{gf}^\pm\right)$, respectively, which are shown in Fig. 1(d) of the main text.

In practical experiments, the external circularly polarized drive field is not ideal and Doppler effect for hot atoms is also considered. The absorption loss for the signal field is determined by the imaginary part of the optical susceptibility

$$\chi_{gf}^\pm = \int_{-\infty}^{\infty} \frac{N(v)\left|\mu_{gf}\right|^2 \sigma_{gf}^\pm(v)}{\varepsilon_0 \hbar \varepsilon_s} dv, \quad (7)$$

where v is the atom velocity along the signal propagation direction, $N(v) = Ne^{-v^2/v_p^2}/(v_p\sqrt{\pi})$ being the Maxwell-Boltzmann velocity distribution, v_p the most probable velocity, N the atomic density, μ_{gf} the transition dipole moment between states $|g\rangle$ and $|f\rangle$, and $\sigma_{gf}^\pm(v) = |g, m_F\rangle\langle f, m_F \pm 1|$ is the atom polarizability due to the probe light for the atom velocity is v. Here the atomic thermal motion should be considered in numerical calculations for the hot atoms, hence the drive fields shifts to $\omega_{d(s)} \pm k_{d(s)}v$, where $+$ ($-$) denotes the same (contrary) input direction for both the drive and signal fields and $k_{d(s)}$ is the wave vector for the drive (signal) laser. For an ideal OIM in a Doppler-broadened

atom ensemble, we have $\chi_{gf}^{-} \propto i/\left(i\Delta_s + \frac{\gamma_{gf}}{2}\right)$ for velocities, and the ensemble optical susceptibility reads

$$\begin{aligned}\chi_{gf}^{\pm} =& \int_{-\infty}^{\infty} \frac{N\left|\mu_{gf}\right|^2 e^{-v^2/v_p^2}}{\varepsilon_0 \hbar \varepsilon_s v_p \sqrt{\pi}} \frac{i}{i\left(\Delta_s + k_s v\right) + \frac{\gamma_{gf}}{2}} dv \\ =& \frac{iN\sqrt{\pi}\left|\mu_{gf}\right|^2 e^{-(i\Delta_s + \gamma_{gf}/2)^2/\left(k_s^2 v_p^2\right)}}{\varepsilon_0 \hbar \varepsilon_s v_p k_s} \\ & \times \left[1 - \mathrm{erf}\left(\frac{i\Delta_s + \gamma_{gf}/2}{v_p k_s}\right)\right]. \end{aligned} \tag{8}$$

For the signal field, the propagation can be calculated by $\partial\varepsilon_s^{\pm}(z)/\partial z = -k_s \mathrm{Im}\left(\chi_{\mathrm{gf}}^{\pm}\right)\varepsilon_{\mathrm{s}}^{\pm}(\mathrm{z})$.

In the cavity-based case, the Hamiltonian for the system can be rewritten as $(\hbar = 1)$:

$$\begin{aligned} H =& \omega_\Omega a_{d\pm}^\dagger a_{d\pm} + \omega_\varepsilon a_{s\pm}^\dagger a_{s\pm} + \sum_{j=1}^{N}\sum_{m_F}\Big\{\omega_g \left|g, m_F\right\rangle\left\langle g, m_F\right|_j + \omega_f\left|f, m_F\right\rangle\left\langle f, m_F\right|_j + \omega_e\left|e, m_F\right\rangle\left\langle e, m_F\right|_j \\ & + g_d\left(\left|e, m_F \pm 1\right\rangle\left\langle g, m_F\right|_j a_{d\pm} + h.c.\right) + g_s\left(\left|f, m_F \pm 1\right\rangle\left\langle g, m_F\right|_j a_{s\pm} + h.c.\right)\Big\} \\ & + i\sqrt{\kappa_{d,ex1}}\Omega_d^{\pm}\left(a_{d\pm}^\dagger e^{-i\omega_d t} - h.c.\right) + i\sqrt{\kappa_{s,ex1}}\varepsilon_s^{\pm}\left(a_{s\pm}^\dagger e^{-i\omega_s t} - h.c.\right), \end{aligned} \tag{9}$$

where ω_Ω and ω_ε are the frequencies of the modes a_d and a_s corresponding to drive and signal modes, respectively, and $g_{d(s)}$ is the single photon coupling rate for the drive/signal field. Considering the decay of both the atom and cavity, the dynamics of the system is governed by the Master equation, which reads

$$\begin{aligned} \frac{d\rho}{dt} =& -i\left[H, \rho\right] + \kappa_d \mathcal{L}\left(a_{d\pm}\right) \\ & + \kappa_s \mathcal{L}\left(a_{s\pm}\right)\sum_{j=1}^{N}\sum_{m_F, m_f'} \gamma_{gf}^{m_F m_F'} \mathcal{L}\left(\left|g, m_F\right\rangle\left\langle f, m_F'\right|_j\right) \\ & + \gamma_{ge}^{m_F m_F'} \mathcal{L}\left(\left|g, m_F\right\rangle\left\langle e, m_F'\right|_j\right). \end{aligned} \tag{10}$$

Here, the formulas are applicable to all emitters with the energy level shown in Fig. 1 of the main context. In current experimental setup, the hot atom vapor is employed, and the analytical solution of the above equation is not practical. Additionally, by taking the uncertainty of environment imperfection and temperature fluctuations, we did not proceed to implement the detailed calculations.

SUPPLEMENTARY NOTE 4 - CAVITY TRANSMISSIONS

For an input to a cavity mode through Port 1, the Hamiltonian can be written as

$$H = \omega_a a^\dagger a + i\sqrt{\kappa_1}(a^\dagger a_{\mathrm{in}} e^{-i\omega_{in} t} - h.c.), \tag{11}$$

where $a_{in} = \sqrt{\frac{P_{\mathrm{in}}}{\hbar\omega_{\mathrm{in}}}}$ is the input amplitude, P_{in} is the input power, ω_a is the resonant frequency of the cavity mode, ω_{in} corresponds to the input signal or drive laser frequency, and κ_1 is the energy decay rate due to the input port. In the rotating frame of input light frequency, the intracavity mode amplitude can be obtained as

$$a = \frac{i\sqrt{\kappa_1}}{-i\Delta - \kappa_{\mathrm{tot}}/2} a_{\mathrm{in}}, \tag{12}$$

with the detuning $\Delta = \omega_a - \omega_{\mathrm{in}}$, and κ_{tot} is the total decay rate.

The traveling-wave cavity composes of four mirrors, including two flat mirrors (M1, M2) and two concave mirrors (M3, M4) as shown in Supplementary Fig. 6(a). In our four-mirror cavity system, we only use the M1 and M2 for input and output, and for both drive and signal. The loss rates of the two ports κ_1 for M1 and κ_2 for M2 can be estimated by

$$e^{-2\pi\kappa_j \tau_{\mathrm{rt}}} = R_j, \tag{13}$$

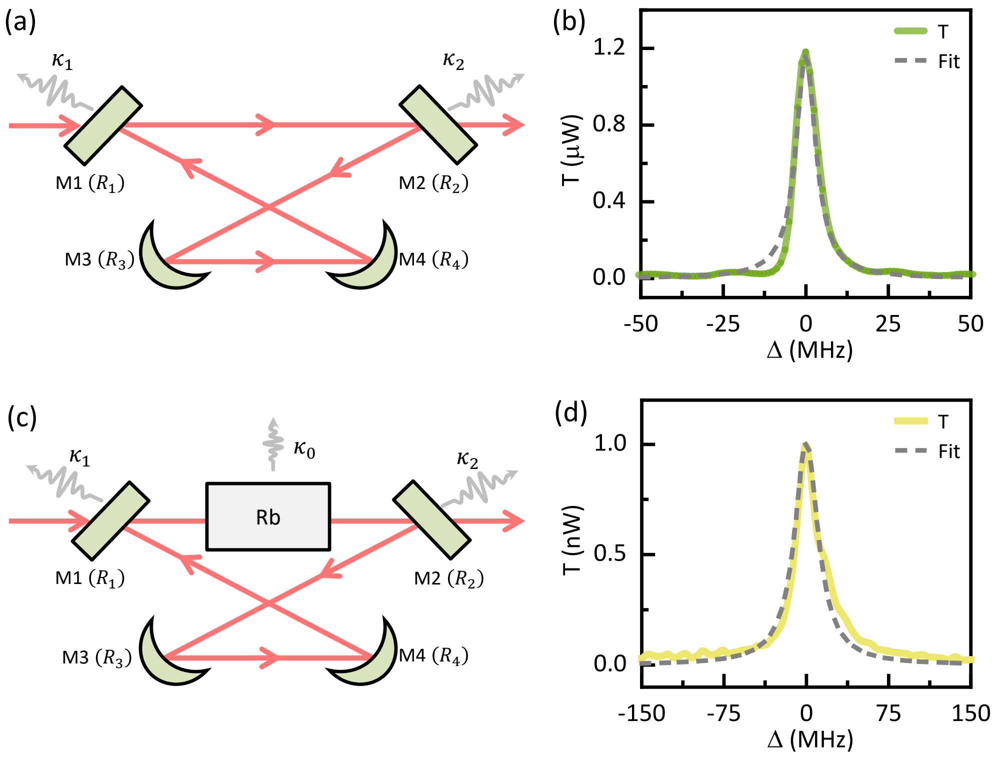

Supplementary Figure 6. **The schematic of theoretical model and the calibration of the traveling-wave cavity parameters.** (a) The scheme of a bare traveling-wave cavity. The cavity composes of four mirrors, where $M_{1,2,3,4}$ corresponds to the mirror with the corresponding reflectivity is $R_{1,2,3,4}$, respectively. M_1 is the input port with the loss $\kappa_{1,1}$ while M_2 is the output port with the loss $\kappa_{1,2}$. (b) The measured transmission of bare cavity (green line) and the fitting curve (gray dashed line) while $P_s = 20\,\mu$W. Based on the fitting result we obtain $\kappa_{1,1} = 7\,$MHz and $\kappa_{1,2} = 0.33\,$MHz, corresponding to the reflectivities of $R_1 \approx 91.8\,\%$ and $R_2 \approx 99.6\,\%$. (c) The scheme of the cavity with a glass vapor cell. The loss due to vapor cell is defined as κ_0. (d) The measured transmission (yellow line) and the fitting result (gray dashed line) while $P_s = 1\,\mu$W. The fitted $\kappa_0 \approx 19\,$MHz.

where $j \in \{1, 2\}$ and $R_{1(2)}$ is the reflectivity of M1(M2), and $\tau_{\rm rt}$ is the cavity round-trip time. In this work, we could ignore the losses of M3 and M4, since they are highly reflective mirrors with $1 - R_{3,4} \ll 1 - R_{1,2}$. In addition, there is also losses introduced by the optical elements placed in the cavity, we denote such an intrinsic energy loss rate of a cavity as κ_0. Then,

$$\kappa_{\rm tot} = \kappa_0 + \kappa_1 + \kappa_2. \tag{14}$$

According to the input-output relation[3], the transmission, i.e. the emission from M2, reads

$$a_{\rm out} = \sqrt{\kappa_2} a = \frac{i\sqrt{\kappa_1 \kappa_2}}{-i\Delta - \kappa_{\rm tot}/2} a_{\rm in}, \tag{15}$$

and the reflected light from the same port $a_{\rm r}$ is

$$a_{\rm r} = -a_{\rm in} + i\sqrt{\kappa_1} a \tag{16}$$

$$= -\frac{-i\Delta - \kappa_{\rm tot}/2 + \kappa_1}{-i\Delta - \kappa_{\rm tot}/2} a_{\rm in}. \tag{17}$$

From the above equations, we could easily obtain the input-output relations for input from M2 by exchanging all subscripts $1 \leftrightarrow 2$. Then, we know that the transmission probing from Port 1 and Port 2 are exactly identical. Therefore, in our experiments, some measurement about the isolation that characterizing the $|a_{\rm out}/a_{\rm in}|^2$ from two opposite directions are actually measured from the same direction. By doing so, we can avoid the calibration of most optical components in the optical setup.

Now, we discuss the basic parameters about the cavity. A typical transmission spectrum of bare cavity is shown in Supplementary Fig. 6(b), with $\kappa_0 = 0$ for an empty cavity system. The green line indicates the experiment result and dashed line corresponds to the theoretical result fitted by

$$P_{\text{out}} = \frac{\kappa_1\kappa_2}{(\kappa_1+\kappa_2)^2/4+\Delta^2}P_{\text{in}}, \tag{18}$$

where input signal power $P_{\text{in}} = 20\,\mu\text{W}$, and the fitting linewidth is 7.33 MHz, i.e. $\kappa_1 + \kappa_2 = 2\pi \times 7.33\,\text{MHz}$. Based on the experimental results of direct signal transmission measurements by a normal incident beam, we find the transmittance of M1 is about 20 times higher than M2, hence we have $\kappa_1 \approx 20\,\kappa_2$. Note that we could not provide an precise measurement of the reflectivities due to the difficulty in absolute calibration of transmission efficiency. Therefore, we can estimate $\kappa_1 \approx 2\pi \times 7\,\text{MHz}$ and $\kappa_2 \approx 2\pi \times 0.33\,\text{MHz}$ and theoretically predict a normalized transmittance of

$$T = \frac{P_{\text{out}}}{P_{\text{in}}} \approx 17.2\,\%, \tag{19}$$

according to Supplementary Eq. (13), with corresponding reflectivities $R_1 \approx 91.8\,\%$ and $R_2 \approx 99.6\,\%$. However, due to the imperfect mode matching between the input beam and the cavity mode field in practical experiments, the measured transmittance reduces to $\sim 5.9\,\%$ comparing to the theoretical expectation. By taking the mode mismatching into account, we rewrite the transmission of the cavity as

$$T = \frac{\kappa_1\kappa_2}{(\kappa_1+\kappa_2)^2/4+\Delta^2} \times \text{A}_{\text{mm}}. \tag{20}$$

where $\text{A}_{\text{mm}} \approx 34\,\%$ represents the mode-matching efficiency in our system.

For the scheme of the system with a glass vapor cell, as shown in Supplementary Fig. 6(c), the glass could induces intracavity scattering and absorption losses κ_0. Then, the transmission of the system becomes

$$T = \frac{\kappa_1\kappa_2}{(\kappa_1+\kappa_2+\kappa_0)^2/4+\Delta^2} \times \text{A}_{\text{mm}}, \tag{21}$$

where input signal power $P_{\text{in}} = 1\,\mu\text{W}$. By measuring and fitting the transmission spectrum, we obtain $\kappa_0 \approx 2\pi \times 19\,\text{MHz}$, as shown in Supplementary Fig. 6(d).

The discussion above indicates that our current experimental demonstration bases on an under-coupled cavity setup since $\kappa_1 \ll \kappa_2 + \kappa_0$, and most of input signal is directly reflected by the cavity and thus induces insertion loss of the device. Therefore, for achieving a useful non-reciprocal device by our scheme, a near-optimal mode matching for input beam and two carefully chosen mirrors ($R_{1,2}$) are important. In the following, we discuss how to achieve the critical coupling for a better isolator performance. The critical coupling ($a_{\text{out}} = 0$) could be achieved for on-resonance light $\Delta = 0$ when the condition

$$\kappa_1 = \kappa_2 + \kappa_0 \tag{22}$$

is satisfied.

In our experiments, the intrinsic loss κ_0 due to the glass atom cell includes atomic absorptive loss $\kappa_{0,1}$ and glass loss $\kappa_{0,2}$. By fitting the linewidth of resonance at a large detuning, as the light absorption due to the atomic transitions is negligible, we estimate $\kappa_{0,1} \approx 2\pi \times 9\,\text{MHz}$, and therefore we have $\kappa_{0,2} \approx 2\pi \times 10\,\text{MHz}$. Since the glass induced loss $\kappa_{0,2}$ could be greatly reduced by improving material and surface treatment, we can assume that the intrinsic loss could be improved to $\widetilde{\kappa_0} = 2\pi \times 9\,\text{MHz}$. It is worth noting that the cavity intrinsic loss could also be reduced by placing the cavity mirrors inside a Rubidium vapor cell [4].

On one hand, for an on-resonant drive field, the intracavity power could be boosted by a factor of

$$\xi = \frac{\widetilde{\kappa_1}}{\left(\widetilde{\kappa_1}+\widetilde{\kappa_2}+\widetilde{\kappa_0}\right)^2}\frac{\left(\kappa_1+\kappa_2+\kappa_0\right)^2}{\kappa_1}. \tag{23}$$

By chosen $\widetilde{\kappa_2} = 2\pi \times 0.1\,\text{MHz}$ and $\widetilde{\kappa_1} = \widetilde{\kappa_2} + \widetilde{\kappa_0} = 2\pi \times 9.1\,\text{MHz}$, i.e. the mirror reflectivities are $R_1 = 89.5\,\%$ and $R_2 = 99.88\%$, we have $\xi \approx 4$, thus the drive power could be reduced by 4 times comparing to the studies in the present work.

On the other hand, by changing the mirror reflectivies for signal's wavelength, the transmission of the signal could be improved to

$$T = \frac{4\widetilde{\kappa_1}\widetilde{\kappa_2}}{\left(\widetilde{\kappa_1}+\widetilde{\kappa_2}+\widetilde{\kappa_0}\right)^2} = 82\% \tag{24}$$

by chosen $\widetilde{\kappa_1} = 2\pi \times 50\,\mathrm{MHz}$ and $\widetilde{\kappa_2} = 2\pi \times 41\,\mathrm{MHz}$, i.e. the mirror reflectivities are $R_1 = 60.7\,\%$ and $R_2 = 54.3\%$, indicating a 1 dB insertion loss and 100 MHz Lorentz-shaped bandwidth of the isolator.

SUPPLEMENTARY NOTE 5 - NOISE ANALYSIS

In this section, we compare the $g^{(2)}(\tau)$ curves of input pseudo-thermal light source to the output curves (Supplementary Fig. 5(c)). The results show that the input and output $g^{(2)}(\tau)$ curves can always match each other very well, which provides a strong evidence that the system is noiseless. For further investigations, we theoretically analyze the model with input thermal noise. The Hamiltonian for the full system can been written as:

$$\begin{aligned} H = &\sum_n \left\{ \Delta_n a_n^\dagger a_n + i\sqrt{\kappa_{1,2}} \left(a_{\mathrm{in}} a_n^\dagger - a_{\mathrm{in}}^\dagger a_n \right) \right\} \\ &+ \sum_n i\sqrt{\kappa_0} \left(a_{\mathrm{noise}} a_n^\dagger - a_{\mathrm{noise}}^\dagger a_n \right) \end{aligned} \tag{25}$$

where n is the number of the modes and $\Delta_n = \omega_n - \omega_p$ is the detuning between the optical mode a_n and the input signal a_{in}. Considering the rate of optical mode, the Langevin equation can be written as follows:

$$\begin{aligned} \frac{da_n}{dt} = &\left(-\frac{\kappa_1 + \kappa_2 + \kappa_0}{2} - i\Delta_n \right) a_n \\ &+ \sqrt{\kappa_2} a_{\mathrm{in}} + \sqrt{\kappa_0} a_{\mathrm{noise}}. \end{aligned} \tag{26}$$

When the system is in the steady state, we have $a_n = \left(\sqrt{\kappa_2} a_{\mathrm{in}} + \sqrt{\kappa_0} a_{\mathrm{noise}} \right) / \left[(\kappa_1 + \kappa_2 + \kappa_0)/2 + i\Delta_n \right]$. Based on the input-output relation $a_{\mathrm{out}} = \sqrt{\kappa_1} \sum_n a_n$, we can obtain

$$a_{\mathrm{out}}^\dagger a_{\mathrm{out}} = \kappa_{1,1} \left(\sum_{m,n} \frac{1}{A_m A_n^*} \right) \left[\kappa_2 a_{\mathrm{in}}^\dagger a_{\mathrm{in}} + \sqrt{\kappa_2 \kappa_0} \left(a_{\mathrm{in}}^\dagger a_{\mathrm{noise}} + a_{\mathrm{noise}}^\dagger a_{\mathrm{in}} \right) + \kappa_0 a_{\mathrm{noise}}^\dagger a_{\mathrm{noise}} \right], \tag{27}$$

$$\begin{aligned} a_{\mathrm{out}}^\dagger a_{\mathrm{out}}^\dagger a_{\mathrm{out}} a_{\mathrm{out}} = &\kappa_{1,1} \left(\sum_{m,n} \frac{1}{A_m A_n^*} \right)^2 \left[\kappa_2^2 a_{\mathrm{in}}^{\dagger 2} a_{\mathrm{in}}^2 + \kappa_2 \kappa_0 a_{\mathrm{in}}^{\dagger 2} a_{\mathrm{noise}}^2 + 2\kappa_2 \sqrt{\kappa_2 \kappa_0} a_{\mathrm{in}}^{\dagger 2} a_{\mathrm{in}} a_{\mathrm{noise}} + \kappa_0 \kappa_2 a_{\mathrm{noise}}^{\dagger 2} a_{\mathrm{in}}^2 \right. \\ &+ \kappa_0^2 a_{\mathrm{noise}}^{\dagger 2} a_{\mathrm{noise}}^2 + 2\sqrt{\kappa_2 \kappa_0} \kappa_0 a_{\mathrm{noise}}^{\dagger 2} a_{\mathrm{in}} a_{\mathrm{noise}} + 2\sqrt{\kappa_{1,2} \kappa_0} \kappa_2 a_{\mathrm{in}}^\dagger a_{\mathrm{noise}}^\dagger a_{\mathrm{in}}^2 \\ &\left. + 2\sqrt{\kappa_2 \kappa_0} \kappa_0 a_{\mathrm{in}}^\dagger a_{\mathrm{noise}}^\dagger a_{\mathrm{noise}}^2 + 4\kappa_2 \kappa_0 a_{\mathrm{in}}^\dagger a_{\mathrm{noise}}^\dagger a_{\mathrm{in}} a_{\mathrm{noise}} \right], \end{aligned} \tag{28}$$

where $A_{m(n)} = (\kappa_1 + \kappa_2 + \kappa_0)/2 + i\Delta_{m(n)}$. Therefore, the normalized correlation function can be written as

$$\begin{aligned} g^2(0) &= \frac{\left\langle a_{\mathrm{out}}^\dagger a_{\mathrm{out}}^\dagger a_{\mathrm{out}} a_{\mathrm{out}} \right\rangle}{\left\langle a_{\mathrm{out}}^\dagger a_{\mathrm{out}} \right\rangle^2} \\ &= \frac{\kappa_{1,2}^2 \left\langle a_{\mathrm{in}}^{\dagger 2} a_{\mathrm{in}}^2 \right\rangle + 2\kappa_0^2 n_{\mathrm{th}}^2 + 4\kappa_2 \kappa_0 n_{\mathrm{th}} \left\langle a_{\mathrm{in}}^\dagger a_{\mathrm{in}} \right\rangle}{\kappa_{1,2}^2 \left\langle a_{\mathrm{in}}^\dagger a_{\mathrm{in}} \right\rangle^2 + \kappa_0^2 n_{\mathrm{th}}^2 + 2\kappa_2 \kappa_0 n_{\mathrm{th}} \left\langle a_{\mathrm{in}}^\dagger a_{\mathrm{in}} \right\rangle}, \end{aligned} \tag{29}$$

where we have assumed that $\langle a_{\mathrm{noise}} \rangle = \left\langle a_{\mathrm{noise}}^\dagger \right\rangle = 0$ and $\left\langle a_{\mathrm{noise}}^\dagger a_{\mathrm{noise}} \right\rangle = \left\langle a_{\mathrm{noise}} a_{\mathrm{noise}}^\dagger \right\rangle - 1 = n_{\mathrm{th}}$ for the thermal noise. It is obvious that we have $g^2(0) = 2$ when the the input a_{in} is also a thermal field. If the input a_{in} is a coherent field α, we have

$$g^2(0) = \frac{\kappa_2^2 |\alpha|^4 + 2\kappa_0^2 n_{\mathrm{th}}^2 + 4\kappa_2 \kappa_0 n_{\mathrm{th}} |\alpha|^2}{\kappa_2^2 |\alpha|^4 + \kappa_0^2 n_{\mathrm{th}}^2 + 2\kappa_2 \kappa_0 n_{\mathrm{th}} |\alpha|^2}. \tag{30}$$

We experimentally measured the the correlation function difference between the input and output signals in Supplementary Fig. 7, which is defined as

$$\begin{aligned} \Delta g^2(\tau) = &\frac{\langle a^\dagger(t) a^\dagger(t+\tau) a(t+\tau) a(t) \rangle_{\mathrm{out}}}{\langle a^\dagger(t) a(t) \rangle \langle a^\dagger(t+\tau) a(t+\tau) \rangle_{\mathrm{out}}} \\ &- \frac{\langle a^\dagger(t) a^\dagger(t+\tau) a(t+\tau) a(t) \rangle_{\mathrm{in}}}{\langle a^\dagger(t) a(t) \rangle \langle a^\dagger(t+\tau) a(t+\tau) \rangle_{\mathrm{in}}}. \end{aligned} \tag{31}$$

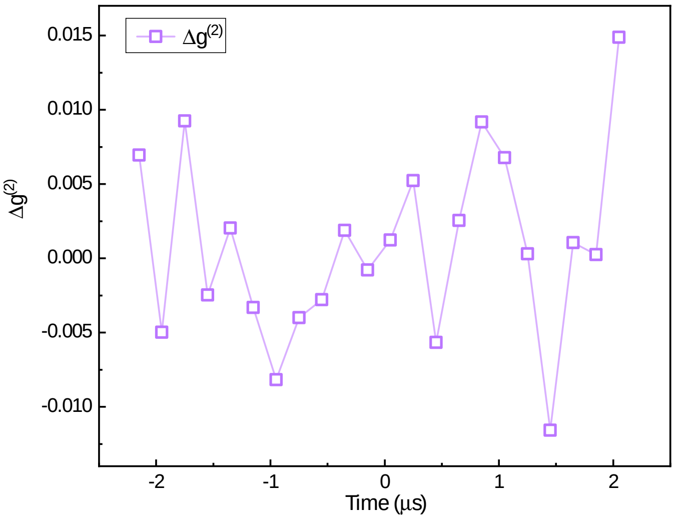

Supplementary Figure 7. The experimental results of the the correlation function differences $[\Delta g^2(\tau)]$ between the input and output signals as a function of the delay time τ.

We fit the experimental data around $\tau \in [-2, 2]$ μs, and obtain the mean value $\overline{\Delta g^2(\tau)} = 0.0008$ and standard deviation $\sigma = 0.00608$. The 95% confidence interval is $[-0.00184, 0.00342]$. In our correlation function measurement experiments, we estimate the intracavity mean photon number about 0.84 for the input coherent signals. By calculating the correlation difference $\Delta g^2(0) = \overline{\Delta g^2(\tau)}$, we can estimate the thermal noise about $n_{\mathrm{th}} = 0.00035$, and the noise-to-signal ratio is about $\eta = \frac{n_{\mathrm{th}}}{\langle a^\dagger a \rangle_{\mathrm{in}}} = 0.04\%$. If we consider the correlation difference $\overline{\Delta g^2(\tau)} + 3\sigma$, we have the noise-to-signal ratio $\eta = 0.9\%$.

---

* These two authors contributed equally to this work.
† zhangpengfei@sxu.edu.cn
‡ chunhua@ustc.edu.cn
§ clzou321@ustc.edu.cn
[1] Hu, X.-X. *et al.* Cavity-enhanced optical controlling based on three-wave mixing in cavity-atom ensemble system. *Opt. Express* **27**, 6660 (2019).
[2] Li, Y. *et al.* Experimental Study on Coherence Time of a Light Field with Single Photon Counting. *Chin. Phys. Lett.* **26**, 074205 (2009).
[3] Walls, D. & Milburn, G. J. *Quantum Optics*. Springer, Berlin (2008).
[4] Cai, B. *et al.* Herriott-cavity-assisted all-optical atomic vector magnetometer. *Phys. Rev. A* **101**, 053436 (2020).
